# Changes of macular sensitivity and morphology after pars plana vitrectomy for macular edema with central retinal vein occlusion: a case series

**DOI:** 10.1186/1471-2415-13-2

**Published:** 2013-02-05

**Authors:** Hidetaka Noma, Tatsuya Mimura, Katsunori Shimada

**Affiliations:** 1 Department of Ophthalmology, Yachiyo Medical Center, Tokyo Women’s Medical University, 477-96, Owada-shinden, Yachiyo, Chiba, 276-8524, Japan; 2 Department of Ophthalmology, Medical Center East, Tokyo Women’s Medical University, Tokyo, Japan; 3 Department of Biostatistics, STATZ Institute Inc, Tokyo, Japan

## Abstract

**Background:**

It is unclear how retinal ischemia influences the changes of visual acuity, macular sensitivity, macular thickness, and macular volume after the performance of pars plana vitrectomy (PPV) for macular edema in patients with central retinal vein occlusion (CRVO).

**Methods:**

Ten patients (10 eyes) with CRVO and macular edema underwent PPV. Retinal ischemia was evaluated from the area of capillary nonperfusion on fluorescein angiography, and the patients were classified into ischemic or nonischemic groups. Microperimetry was performed with a Micro Perimeter 1. Macular thickness and volume were measured by optical coherence tomography.

**Results:**

In both groups, the mean macular thickness within the central 4°, 10°, and 20° fields decreased significantly from baseline to 3 and 6 months after PPV (all P < 0.05). In the ischemic group, the mean macular sensitivity within the central 4°, 10°, and 20° fields increased from baseline to 3 and 6 months after PPV, but no significant difference was observed.

**Conclusions:**

These results suggest that PPV may be effective for improving macular thickness, volume, and sensitivity in patients with macular edema secondary to ischemic CRVO, although there was no significant difference.

## Background

Macular edema is the most vision-threatening complication of central retinal vein occlusion (CRVO). Vascular endothelial growth factor (VEGF) is thought to have an important role in the pathogenesis of macular edema [1], and anti-VEGF therapy has been shown to improve macular edema over both the short-term and the long-term [2,3]. In addition to intravitreal injection of anti-VEGF agents [4], macular edema associated with CRVO can be treated by injection of triamcinolone acetonide [5] or by pars plana vitrectomy (PPV) [6]. These methods have all been reported to improve both visual acuity and macular edema. However, evaluation of visual function from visual acuity alone is problematic because macular edema also involves the larger macula area beyond the fovea in most CRVO patients and measurement of visual acuity only assesses foveal function. Thus, a reproducible method of measuring retinal function is needed to more accurately assess the response of macular edema to treatment.

The Micro Perimeter 1 (MP-1) is an instrument that combines digital fundus imaging with automated perimetry [7]. Thus, it allows evaluation of both the fovea and the entire macular region, unlike measurement of visual acuity. Recently, we reported that retinal thickness and retinal volume are correlated with both retinal sensitivity and best-corrected visual acuity (BCVA) in CRVO patients who have macular edema [8]. This suggests that BCVA may be an inadequate parameter for evaluating the response of macular edema to treatment, while the functional prognosis may be better assessed by measuring the retinal sensitivity of the fovea and the entire macular region with the MP-1. In addition, the visual prognosis of CRVO patients with macular edema treated by intravitreal bevacizumab was reported to be influenced by retinal ischemia [9]. However, little is known about the influence of retinal ischemia on retinal sensitivity after performance of PPV for macular edema in CRVO patients. Therefore, we evaluated the influence of retinal ischemia on changes of visual acuity, macular sensitivity, macular thickness, and macular volume after PPV in CRVO patients with macular edema.

## Methods

### Subjects

This study was approved by the institutional review boards of Tokyo Women’s Medical University and adhered to the tenets of the Declaration of Helsinki. Written informed consent was obtained from each patient. Pars plana vitrectomy was performed because it has been reported that macular edema and visual acuity can be improved by this procedure in CRVO patients [6]. We retrospectively studied 10 eyes of 10 patients with macular edema with CRVO who were treated with PPV. Ten patients (mean age: 71.6 ± 7.2 years; 7 women and 3 men) were included in this uncontrolled study conducted at the Department of Ophthalmology of Tokyo Women’s Medical University between May 2009 and July 2011. Patients were diagnosed as having hypertension if the systolic blood pressure was ≥140 mm Hg and diastolic blood pressure was >90 mm Hg, or if the systolic pressure was ≥140 mm Hg at one examination and the diastolic pressure was ≥90 mm Hg on a different day, or if the patient was already taking antihypertensive medication. A diagnosis of hyperlipidemia was based on a total cholesterol≥240 mg/dL, triglycerides≥160 mg/dL, low-density lipoprotein cholesterol≥130 mg/dL, or use of cholesterol-lowering medication.

The indications for treatment of macular edema with PPV were: (1) cystoid macular edema, (2) a foveal thickness greater than 300 μm, and (3) best-corrected visual acuity worse than 20/50. The exclusion criteria were (1) previous ocular surgery, (2) diabetes mellitus with diabetic retinopathy, (3) previous macular laser photocoagulation, (4) previous intravitreal injection of anti-VEGF agents or triamcinolone acetonide, (5) a history of ocular inflammation, (6) marked retinal hemorrhage (including macular bleeding involving the intrafoveal or subfoveal spaces), (7) coexisting ocular disease (i.e., epiretinal membrane or glaucoma), and (8) retreatment during the 6-month follow-up period.

All patients had undergone a comprehensive ophthalmologic examination, including best-corrected visual acuity measurement, intraocular pressure determination, indirect ophthalmoscopy, and slit-lamp biomicroscopy with a contact lens before and at 3, and 6 months after treatment. In addition, retinal sensitivity was investigated by microperimetry, and retinal thickness and retinal volume were measured by OCT.

### Surgical procedure

Ten patients received pars plana vitrectomy under local anaesthesia. When PPV was done, all epiretinal materials, the residual cortex, and the posterior hyaloid were removed from the retinal surface around the macula as completely as possible with the assistance of triamcinolone acetonide (the minimum necessary volume was injected and then was removed as thoroughly as possible at the end of surgery, because it can influence cystoid macular edema). Internal limiting membrane (ILM) peeling was not done because peeling-related retinal injury can have a variable effect on retinal sensitivity, which could have caused bias in this study. Intraoperative scatter laser photocoagulation to the ischemic region of the retina was not done. During surgery, iatrogenic peripheral retinal tears did not occur in any of the patients. Concurrent phacoemulsification with intraocular lens insertion into the bag was performed. All of the patients who had cataract surgery were classified into Emery-Little grades I or II according to the new World Health Organization Simplified Cataract Grading System. All patients were followed up for at least 6 months postoperatively. Neovascular glaucoma and iris neovasculariation were not detected in any of the patients after follow up for six months, so trabeculectomy or tube shunt surgery was not performed up to that time.

### Fundus examination

As baseline screening, patients underwent ophthalmoscopy and biomicroscopic examination using a slit-lamp with a fundus contact lens. They also underwent standard fundus color photography and fluorescein angiography, which was performed with a Topcon TRC-50EX fundus camera, an image-net system (Tokyo Optical Co. Ltd., Japan), and a preset lens with a slit-lamp.

A masked grader independently assessed ischemic retinal vascular occlusion on the fluorescein angiograms by measuring the ischemic area of the retina with the public domain Scion Image program, as reported previously [10-12]. On digital photographs of the fundus, the optic disc was outlined with a cursor and then its area was measured, as was also done for the nonperfused area of the retina. Then the nonperfused area was divided by the disc area to calculate the severity of retinal ischemia. If the non-perfused area divided by the disc area gave a value of 10 or more, this was defined as indicating the presence of retinal ischemia (ischemic CRVO group) [13-15].

### Measurement of BCVA

Each patient underwent measurement of best-corrected visual acuity (BCVA) with an SC-2000 System chart (Nidek, Gamagori, Japan). BCVA was measured in decimal units on a Landolt chart by the orthopticists. The chart brightness was set at 80–320 cd/m^2^, and chart contrast was more than 74%. The results were converted to the logarithm of the minimum angle of resolution scale (log MAR).

### Measurement of optical coherence tomography

OCT was performed with an instrument from Zeiss-Humphrey Ophthalmic Systems (Zeiss Stratus OCT3, Carl Zeiss Meditec, Dublin, CA, USA) to measure the foveal thickness. At each visit, all patients underwent Stratus OCT examination in the vertical cross-section with the instrument centered on the fovea and in the fast macular thickness mode. On these views, retinal thickness was defined as the distance between the inner surface of the neurosensory retina and the retinal pigment epithelium. Foveal thickness was calculated as the average retinal thickness within a circle of 500-μm radius centered on the fovea. A retinal thickness map and retinal volume map were obtained by scanning 6×6 mm (20°×20°) areas of the macular region, which was divided into the following nine subfields: 1) fovea, 2) superior inner macula, 3) nasal inner macula, 4) inferior inner macula, 5) temporal inner macula, 6) superior outer macula, 7) nasal outer macula, 8) inferior outer macula, and 9) temporal outer macula [8]. The diameters of the central, inner, and outer circles were 1, 3, and 6 mm, respectively. In each region, measurement of retinal thickness and volume was automatically performed by computer software. The mean macular thickness at the one subfield (fovea) covering the central 1×1 mm (4°×4°), at five subfields (fovea, superior inner, nasal inner, inferior inner, and temporal inner) covering the central 3×3 mm (10°×10°), and at all nine subfields covering the entire central 6×6 mm (20°×20°) were thus determined.

### Functional mapping by microperimetry

Microperimetry with the MP-1 (Nidek, Gamagori, Japan) is performed using an infrared fundus camera with a liquid crystal display controlled by special software. The MP-1 software contains an automatic tracking system for fundus movements; this evaluates every acquired frame for shifts in the x and y directions of the fundus with respect to a reference frame obtained by an infrared camera at the beginning of the examination. Each patient underwent fundus-monitored microperimetry with the MP-1 system (Nidek, Gamagori, Japan). Its software performs automatic tracking of fundus movements and evaluates every frame acquired for fundus shift in the x and y directions relative to a reference frame obtained with an infrared camera at the beginning of the examination. Microperimetry settings were identical for all examinations: Goldmann III stimuli were presented in random order according to a 4-2-1 double staircase strategy. The stimulus intensity ranged from 0 to 20 decibels (dB) (0 dB corresponded to the strongest signal intensity of 127 cd/m^2^) in 1-dB steps, and the duration of each stimulus was 200 ms. The fixation target was varied in size according to the patient’s visual acuity. Retinal sensitivity maps were obtained by using the macula 20 degrees program of the MP-1. During the examination, background illumination was set at 1.27 cd/m^2^. Mean retinal sensitivity was calculated from the sensitivity for each of nine subfields on the retinal map generated by OCT. The mean macular sensitivities at the five locations covering the central 4° field, at 29 locations covering the central 10° field (five subfields; fovea, superior inner, nasal inner, inferior inner, and temporal inner), and at the 57 locations covering the entire central 20° field (all nine subfields) were thus determined.

### Statistical analysis

All analyses were performed with SAS System 9.1 software (SAS Institute Inc., Cary, North Carolina, USA). Results are presented as the mean ± SD or as the frequency. One-way repeated measures analysis of variance (ANOVA) was used to evaluate the changes of visual acuity, macular sensitivity, macular thickness, and macular volume. Two-tailed P values of less than 0.05 were considered to indicate statistical significance.

## Results

The characteristics of the ischemic and nonischemic groups are summarized in Table 1. Among the 10 patients with CRVO, 6 were assigned to the ischemic group and 4 to the nonischemic group. There were no significant differences between the two groups with regard to the mean age, female/male ratio, prevalence of hypertension, prevalence of hyperlipidemia, and duration of CRVO (P = 0.133, P = 0.500, P = 0.778, P = 0.190, and P = 0.061, respectively).

**Table 1:** Baseline clinical features of the ischemic and nonischemic groups

**Findings**	**Ischemic (N = 6)**	**Nonischemic (N = 4)**	**P value**
Age (years)	72.6±7.3^‡^	70.0±7.7^‡^	0.133
Gender (female/male)	5/1	2/2	0.500
Hypertension	4	3	0.778
Systolic blood pressure (mmHg)	137±23^‡^	133±14^‡^	0.746
Diastolic blood pressure (mmHg)	78±6^‡^	77±11^‡^	0.845
Hyperlipidemia	1	3	0.190
Duration of CRVO (months)	4.3±2.3^‡^	8.5±3.9^‡^	0.061
Visual acuity (log MAR)	1.26 ± 0.41^‡^ (range; 1.0 to 2.0)	0.59 ± 0.38^‡^ (range; 0.30 to 1.15)	0.031
Cataract (Emery-Little grade)	1.66 ± 0.40^‡^	1.25 ± 0.28^‡^	0.117

*CRVO* = central retinal vein occlusion; *log MAR* = logarithm of the minimum angle of resolution scale;

^‡^Mean ± standard deviation (SD).

**Figure 1:**
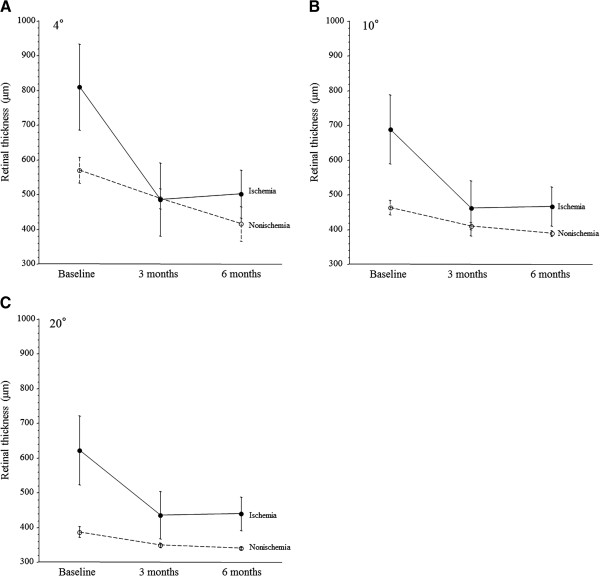
**Changes of the mean macular thickness (A-C) after pars plana vitrectomy (PPV) in CRVO patients with macular edema.** In the ischemic group, the mean macular thickness within the central 4°, 10°, and 20° fields decreased significantly from baseline to 3 and 6 months after PPV (P = 0.007, P = 0.012, and P = 0.040, respectively). In the nonischemic group, the mean macular thickness within the central 4°, 10°, and 20° fields also decreased significantly from baseline to 3 and 6 months after PPV (P = 0.002, P = 0.007, and P = 0.030, respectively).

In the ischemic group, the mean macular thickness within the central 4°, 10°, and 20° fields showed a significant decrease from baseline to 3 and 6 months after PPV (P = 0.007, P = 0.012, and P = 0.040, respectively) (Figure 1A-C). In the nonischemic group, the mean macular thickness within the central 4°, 10°, and 20° fields also showed a significant decrease from baseline to 3 and 6 months after PPV (P = 0.002, P = 0.007, and P = 0.030, respectively) (Figure 1A-C).

**Figure 2:**
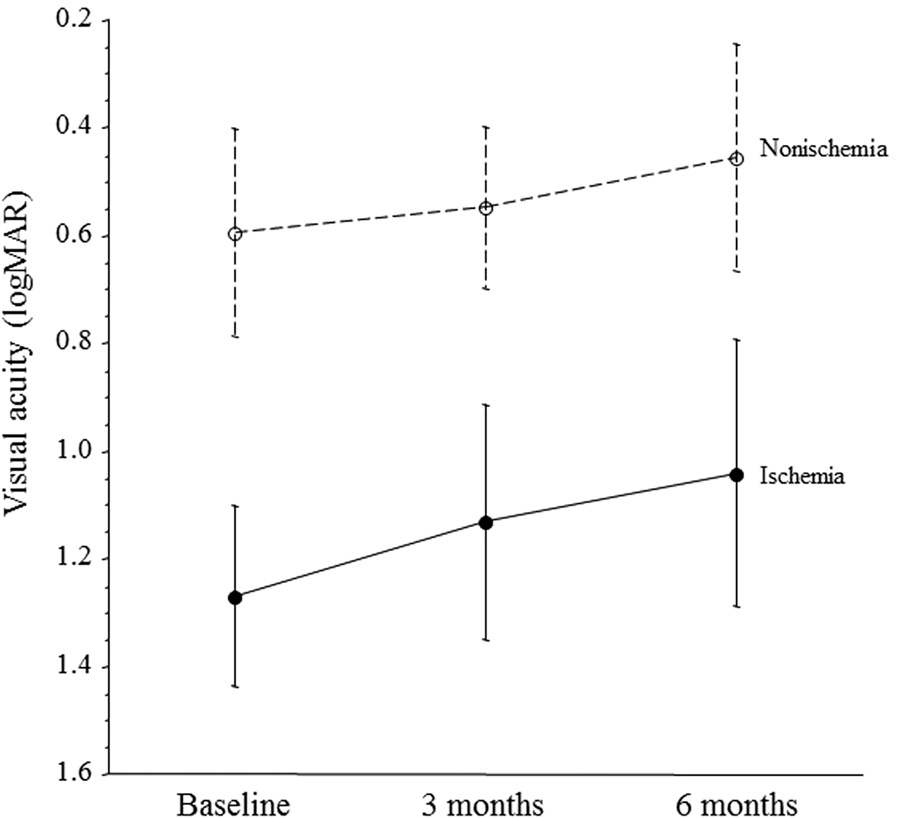
**Changes of mean visual acuity after pars plana vitrectomy (PPV) in CRVO patients with macular edema.** In the ischemic group, visual acuity improved from baseline to 3 and 6 months after PPV, although not significantly (P = 0.569). Likewise, the nonischemic group showed improvement of visual acuity from baseline to 3 and 6 months after PPV, although it was not significant (P = 0.359).

The visual acuity of the ischemic group improved from baseline to 3 and 6 months after PPV, although not significantly (P = 0.569) (Figure 2). Likewise, the visual acuity of the nonischemic group showed some improvement from baseline to 3 and 6 months after PPV, although it was also not significant (P = 0.359) (Figure 2).

**Figure 3:**
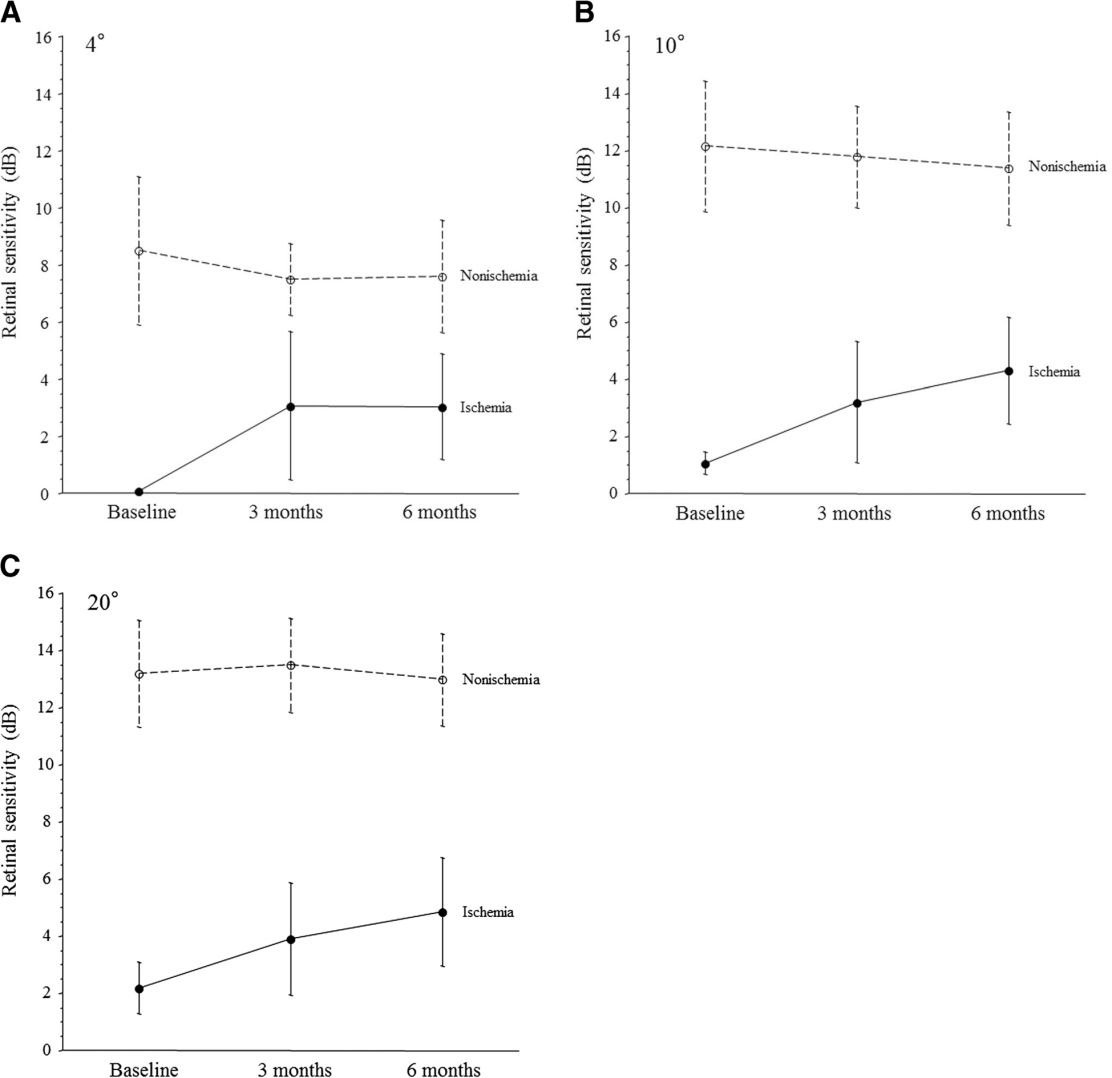
**Changes of mean macular sensitivity (A-C) after pars plana vitrectomy (PPV) in CRVO patients with macular edema.** In the ischemic group, the mean macular sensitivity within the central 4°, 10°, and 20° fields increased from baseline to 3 and 6 months after PPV, although not significantly (P = 0.233, P = 0.179, and P = 0.247, respectively). In the nonischemic group, however, the mean macular sensitivity within the central 4°, 10°, and 20° fields was not increased from baseline to 3 and 6 months after PPV (P = 0.620, P = 0.226, and P = 0.321, respectively).

In the ischemic group, the mean macular sensitivity within the central 4°, 10°, and 20° fields increased from baseline to 3 and 6 months after PPV, although the changes were not significant (P = 0.233, P = 0.179, and P = 0.247, respectively) (Figure 3A-C). In the nonischemic group, however, mean macular sensitivity within the central 4°, 10°, and 20° fields was not increased from baseline to 3 and 6 months after PPV (P = 0.620, P = 0.226, and P = 0.321, respectively) (Figure 3A-C).

Improvement of visual acuity and macular sensitivity was calculated by subtracting the postoperative value from the preoperative value. Improvement of macular edema was evaluated by calculating the percent change of macular edema (%ΔME) as follows: %ΔME = (ME_pr_−ME_po_)/ME_pr_ × 100 = (1−ME_po_/ME_pr_) × 100, where ME_pr_ and ME_po_ are the macular thickness and volume before vitrectomy and 6 months after surgery, respectively. It was found that the duration of CRVO had no significant influence on the improvement of visual acuity (ρ = 0.18, P = 0.619); the improvement of macular sensitivity within the central 4° field (ρ = 0.20, P = 0.579), 10° field (ρ = 0.31, P = 0.376), or 20° field (ρ = 0.33, P = 0.346); the improvement of macular thickness within the central 4° field (ρ = −0.09, P = 0.806), 10° field (ρ = −0.20, P = 0.578), or 20° field (ρ = −0.22, P = 0.538); and the improvement of macular volume within the central 4° d (ρ = −0.16, P = 0.665), 10° field (ρ = −0.17, P = 0.634), or 20° field (ρ = −0.20, P = 0.580).

## Discussion

The present study demonstrated that the mean macular thickness within the central 4°, 10°, and 20° fields decreased significantly from before PPV to 3 and 6 months after PPV in patients with either ischemic or nonischemic CRVO. It has been reported that an increase of oxygen tension in the inner retina could be an important effect of PPV [16,17]. If the retinal oxygen tension increases after PPV, macular edema would decrease for the following two reasons: 1) an increase of oxygen tension would reduce VEGF production and decrease vascular permeability to reduce edema, and 2) an increase of oxygen tension would alleviate autoregulatory arteriolar vasoconstriction and thus lower the hydrostatic pressure in the retinal capillaries and venules, so that decreased water flux from the vascular compartment to the tissue compartment would reduce edema according to Starling’s law. Furthermore, the increase of oxygen tension in the inner retina seems to persist over the long term after PPV, so surgery may have a significant influence on macular edema. We previously reported that the vitreous fluid levels of various inflammatory factors were elevated in CRVO patients [1,18]. If PPV reduces the intraocular levels of inflammatory factors, this may be another mechanism by which surgery improves macular edema in CRVO patients. Such a mechanism is supported by our report that a high vitreous VEGF level was associated with greater improvement of macular edema after PPV [19].

Regarding macular sensitivity, we found that the mean macular sensitivity within the central 4°, 10°, and 20° fields was increased from baseline to 3 and 6 months after PPV in the ischemic group (although not significantly), while it did not increase in the nonischemic group. Fluid currents carrying oxygen from well-perfused to ischemic areas of the retina may be increased by PPV, improving oxygenation of the ischemic inner retina and raising the local oxygen tension [16,17]. An increase of oxygen tension in the inner retina after PPV could reverse functional impairment of photoreceptor cells, resulting in improvement of macular sensitivity. In summary, our results suggest that PPV may be more effective for improving retinal sensitivity over the larger macular area in patients with ischemic CRVO than in those with nonischemic CRVO.

Anti-VEGF therapy was recently reported to improve macular edema in CRVO patients, but most of the subjects had nonischemic CRVO [4]. In another study, anti-VEGF therapy was found to be effective for macular edema in patients with nonischemic retinal vein occlusion, but not in those with ischemic retinal vein occlusion [2]. Taken together, these findings and our results suggest that if CRVO patients have retinal ischemia, a new strategy of performing PPV if anti-VEGF therapy fails could be considered from the viewpoint of improving macular sensitivity. However, the present study had several limitations, including a short follow-up period, small sample size, and no control group. Accordingly, a randomized, prospective clinical trial of these therapies would be required to confirm the efficacy of PPV for macular edema in patients with ischemic CRVO.

## Conclusions

Macular sensitivity within the central 4°, 10°, and 20° fields was improved from baseline to 3 and 6 months after PPV in patients with ischemic CRVO, but no significant difference was observed. These results suggest that PPV may be effective for improving macular edema and macular sensitivity in patients with ischemic CRVO.

## Competing interests

The authors declare that they have no competing interests.

## Authors’ contributions

HN was involved in the design and conduct of the study. Collection and management of the data were done by HN, and KS, while analysis and interpretation of the data were performed by HN, TM, and KS. Preparation of the first draft of the manuscript was done by HN and review and approval of the manuscript was performed by TM and KS. All authors read and approved the final manuscript.

## Pre-publication history

The pre-publication history for this paper can be accessed here:

http://www.biomedcentral.com/1471-2415/13/2/prepub
